# New World Ocular Dirofilariasis Caused by *Dirofilaria repens* Infection, United States

**DOI:** 10.3201/eid3207.251596

**Published:** 2026-07

**Authors:** Ben J. Glasgow, Mackenzie Collins, Luke Helminiak, Joshua A. Lieberman, Blaine A. Mathison, Shangxin Yang

**Affiliations:** University of California Los Angeles David Geffen School of Medicine, Los Angeles, California, USA (B.J. Glasgow, M. Collins, L. Helminiak, S. Yang); University of Washington, Seattle, Washington, USA (J.A. Lieberman); ARUP Laboratories and University of Utah, Salt Lake City, Utah, USA (B.A. Mathison)

**Keywords:** Ocular dirofilariasis, *Dirofilaria repens*, eyelid infection, nematode, parasites, parasitic infection, zoonoses, California, United States

## Abstract

We describe an infection caused by *Dirofilaria repens* nematodes in California, USA. A firm nodule developed after an insect bite on a patient’s eyelid. Excision with morphologic and molecular analysis confirmed *D. repens* infection. Our findings confirm the necessity of both molecular and histological studies to identify nematode infections.

Dirofilariasis is caused by *Dirofilaria* (family Onchocercidae) nematodes. Old World infections are commonly caused by *Dirofilaria repens* nematodes. New World infections are generally caused by species other than *D. repens*, such as *D. immitis*, *D. tenuis*, *D. subdermata*, *D. striata*, and *D. ursi* (*1*). Canids, felids, and raccoons are the definitive hosts for most zoonotic infections, and mosquitoes serve as intermediate vectors. In humans, ocular dirofilariasis, which includes eyelid, subconjunctival, orbital, and intraocular infections, accounts for <35% of all cases (*2*). The eyelid and orbit are the sites of ≈42% of ocular dirofilariasis cases (*3*). The species causing ocular dirofilariasis have distinct geographic associations (*3*). We describe ocular dirofilariasis caused by *D. repens* nematodes in California, USA.

A 74-year-old man from California was bitten by an insect on his left lower eyelid. Initially, he experienced transient pain, swelling, and weeping at the wound. Six weeks later, his dermatologist noted an 8-mm diameter, firm, nontender subcutaneous nodule at this site. The patient had no medical history or recent travel of note. After referral to an ophthalmologist, magnetic resonance imaging of the orbits confirmed a well-circumscribed cystic lesion on the eyelid (Figure 1, panel A). The mass persisted for 5 months, and an excisional biopsy was performed. The mass was dissected from closely adherent surrounding tissue and submitted to pathology in formalin. The patient was asymptomatic 6 months after the surgery.

**Figure 1:**
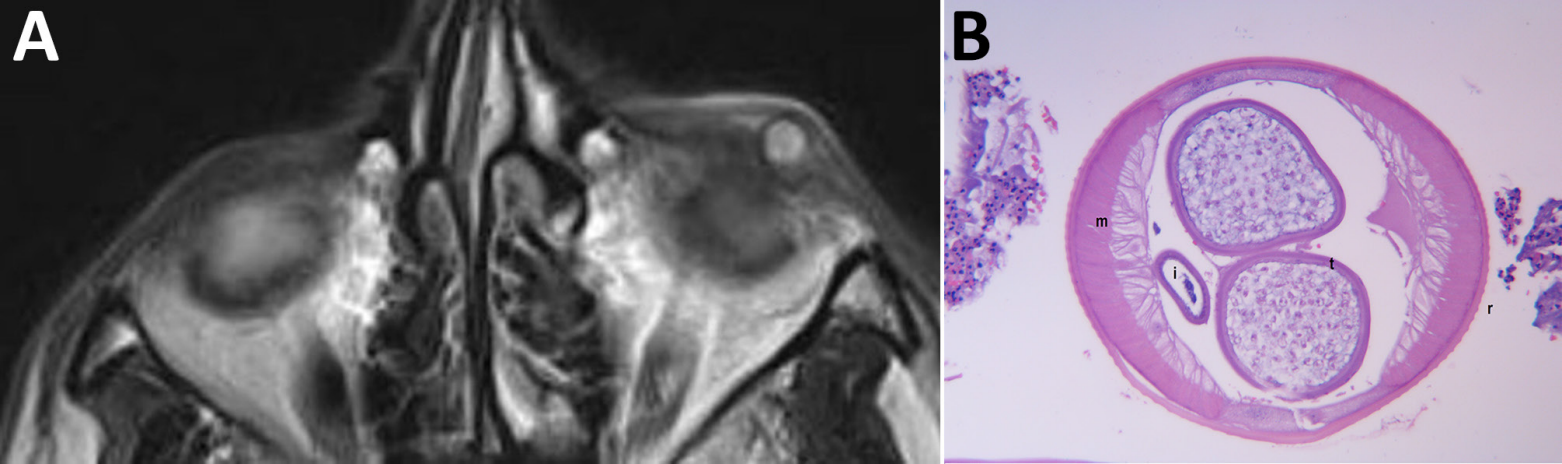
Images from patient with ocular dirofilariasis caused by *Dirofilaria repens* in California, USA. A) Axial plane of T-2–weighted orbital magnetic resonance imaging revealing a well circumscribed cystic lesion in the left lower eyelid. The center of the lesion is hyperintense and circumscribed by a hypointense signal. The interpretation was a benign inflammatory lesion. B) Histopathologic image from hematoxylin and eosin–stained slide that reveals a 500-micron diameter cross section of a nematode with a thick cuticular wall featuring external cuticular ridges (indicated by r) and vertically oriented muscle (indicated by m) extending toward the internal cavity (coelomyarian) that are numerous per quadrant (polymyarian), as well as paired reproductive tubes (indicated by t), and simple intestine (indicated by i).

The tissue was processed routinely and sectioned after paraffin embedding. Microscopy revealed a parasite surrounded by fibrosis with marked chronic inflammation. The cross section of the parasite revealed features of a nematode consistent with *Dirofilaria* sp. (Figure 1, panel B).

The formalin-fixed paraffin-embedded tissue was sent to the University of Washington Reference Laboratories (Seattle, WA, USA) for species identification, where we conducted broad-range 28S and internal transcribed spacer (ITS) rDNA PCR and sequencing (*4*). We detected *D*. *repens* DNA. We conducted an in-house ITS-based targeted next-generation sequencing assay on the same formalin-fixed paraffin-embedded tissue, as previously described (*5*), confirming the species as *D. repens*. The ITS sequence matched reference sequences from GenBank with 100% pairwise identity. In addition, we conducted shotgun sequencing by using Illumina Miseq (Illumina, https://www.illumina.com) to acquire more genetic information about the parasite. We submitted raw sequence reads to Chan Zuckerberg ID (https://czid.org) for metagenomic analysis, gathering reads aligning to *Dirofilaria* species. We generated consensus sequence of the COX1 gene by mapping the aligned reads to a *D. repens* mitochondrial DNA reference (GenBank accession no. KX265049). We conducted phylogenetic analysis on the basis of all 3 marker genes (ITS, 28S, and COX1), which further confirmed the species identification (Figure 2).

**Figure 2:**
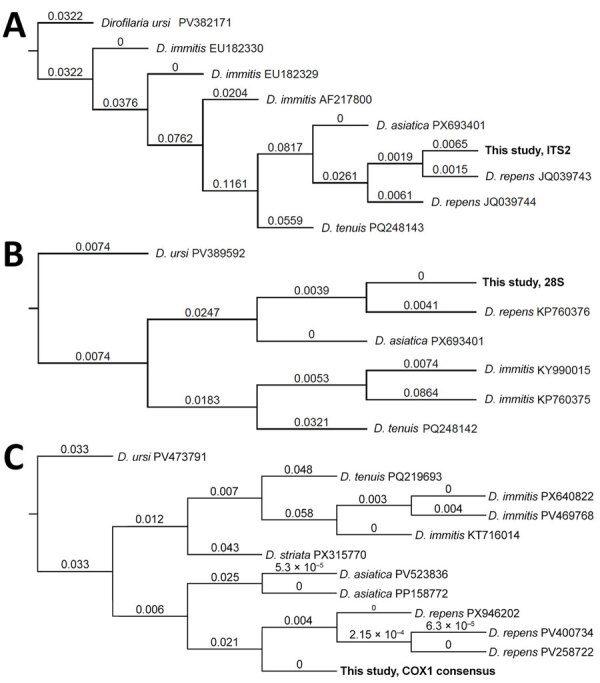
*Dirofilaria repens* phylogenetic trees from study of ocular dirofilariasis in California, USA. A) ITS gene sequences; B) 28S gene sequences; C) COX1 gene sequences. All 3 marker gene comparisons confirmed the study sample belonged to *D. repens*. Genbank accession numbers are indicated. Branch numbers indicate nucleotide substitutions per site. ITS, internal transcribed spacer.

The identification of *D. repens* infection in the United States is noteworthy because of the parasite’s previous absence. Microfilariae of *D. repens* have been reported in ring-tailed coati in Brazil and Chile (*6*,*7*). A nationwide survey of domestic hosts in the United States identified *D. immitis* parasites in 6.3% of 1,080 dogs and 0.3% of 1,254 cats, but all samples were negative for *D. repens* parasites (*8*). The case-patient’s chronology of infection is consistent with the development of infective third-state larvae into juvenile worms, ≈50 days (*2*). Infection from a domestic host is likely. The recently increased population of *Aedes* mosquitoes in southern California might have contributed, but the lack of available *D. repens* surveys in wildlife hosts from California hampers conclusive findings.

Most cases of ocular dirofilariasis and all previous cases of dirofilariasis infections of the eyelid reported in the United States were attributed to *D. tenuis* infection (*3*). *D. repens* is the most common infection of the eyelid in the Old World, with only rare cases of *D. asiatica* infection reported (*9*). Various species share common characteristics including a multilayered cuticle, coelomyarian or polymyarian muscle cells, simple intestine, paired sterile reproductive tubes, and internal lateral ridges (*3*). *D. tenuis* and *D. repens* nematodes both have external ridges, which *D. immitis* nematodes lack. For cases in which speciation was on the basis of morphologic assessment alone, we cannot exclude possible errors.

Most previous *D. repens* eyelid infections were localized and did not result in patent infection. Young adult nematodes are usually seen in the human, an unsuitable or accidental host. Of note, rare exceptions exist in which the worm was able to mature subcutaneously and produce microfilariae (*10*).

Eyelid dirofilariasis frequently masquerades as other entities, such as a neoplasm, chalazion, or benign cyst. In this case, a chalazion was suspected. Prior cases in the literature of dirofilariasis have shown similar cystic changes with enhancement on magnetic resonance imaging (Figure 1). Other parasitic infections, such as cysticercosis, leishmaniasis, and rarely loiasis, can produce single eyelid cysts.

We report a case of *D. repens* human infection in California, USA. Suspicion of this disorder is predicated on a careful history of the environment with mosquitos, raccoons, canids, or felids. The identification of *D*. *repens* nematodes in the United States warrants continued surveillance. Careful histologic examination and molecular studies are critical for parasite identification. 

Appendix Additional information about new world ocular dirofilariasis caused by *Dirofilaria repens.*
