# Supplementary material for: New World Ocular Dirofilariasis Caused by Dirofilaria repens Infection, United States
Source: Emerg Infect Dis. 2026 Jul;32(7):1193–5. doi: 10.3201/eid3207.251596 (PMC13322429; doi:10.3201/eid3207.251596)
Supplement: Appendix — Additional information about new world ocular dirofilariasis caused by Dirofilaria repens. [file 25-1596-Techapp-s1.pdf]

Article DOI: https://doi.org/10.3201/eid3207.251596

EID cannot ensure accessibility for supplementary materials supplied by authors. Readers who have difficulty accessing supplementary content should contact the authors for assistance.

New World Ocular Dirofilariasis Caused by *Dirofilaria repens* Infection, United States

Appendix

Supplemental Methods

DNA extraction yield and quality

Three separate FFPE DNA extractions were performed, including one at a reference laboratory (University of Washington, UW) for broad range PCR and sequencing with an unknown kit. The other two extractions were performed in house, where DNA was extracted from formalin-fixed paraffin-embedded (FFPE) tissue using the QIAamp DSP DNA FFPE Tissue Kit (QIAGEN, Hilden, Germany, cat# 60404) following the manufacturer's protocol, including CitriSolv-based deparaffinization, proteinase K digestion, and heat-mediated reversal of formalin crosslinking. DNA quantity was assessedwith Qubit fluorometry.

Extractions yielded a total of 1 ug and 1.8 ug. For FFPE-derived material, DNA is expected to be fragmented, with amplifiable fragment sizes typically in the range of ~150–500 bp, consistent with prior reports for FFPE tissues. Despite fragmentation, DNA was sufficient for downstream PCR and sequencing. Cleaned-up PCR product was analyzed with the Agilent 2100 Bioanalyzer High Sensitivity DNA assay. The sample demonstrated fragment distribution between ~200–1000 bp, with an average length of 434 bp. This region included 93% of total DNA in the sample at a concentration of 7.4 ng/uL with 31.4% CV for fragment distribution, compatible for short-read sequencing.

PCR amplification

1. At University of Washington, broad-range PCR targeting the 28S gene was performed. In-house targeted ITS PCR and sequencing workflow has been validated in our laboratory, however ITS sequence was not sufficient for definitive species identification. A final sequencing

run was performed on a separate FFPE tissue extraction and shotgun sequencing produced sufficient coverage over the *Dirofilaria* mitochondrial genome to generate a COX1 consensus sequence most closely aligned to *D. repens* (uploaded to GenBank with accession PZ357936). The PCR amplification success rate was high, with over 95% for the 1st time, and 100% after repeat.

2. PCR cleanup was bead-based. AMPure XP Beads (Beckman Coulter, Brea, CA). PCR products were incubated with beads for 5 minutes at a 0.8x DNA: bead volume ratio and washed twice on a magnet with 200uL of 80% EtOH. The pellet was dried and resuspended in 35uL of nuclease-free H2O for 2 minutes off of the magnet. Eluate was separated from the beads, collected, and taken into library preparation.

Contamination control measures

To minimize contamination, multiple precautions were implemented. These include the physical separation of pre- and post-PCR workflows and the use of dedicated equipment and consumables for DNA extraction, PCR setup, and library prep. Use of aerosol-resistant filtered tips, surface decontamination, along with the inclusion of extraction and PCR no-template controls (NTCs) provided robust contamination checks. Additionally, sequence data were evaluated for unexpected hits or mixed signals; no evidence of cross-sample or exogenous DNA contamination was observed.

Downstream bioinformatic filtering was applied to remove low-quality reads and potential contaminants. Only high-confidence reads were retained for analysis. Reads from shotgun sequencing were uploaded to CZID to selectively obtain sequences aligning to *Dirofilaria* species and deplete host reads. Targeted and shotgun sequences also identified the presence of *Wolbachia* genetic material (*D. repens* endosymbiont, accession# AJ276500) uniquely in our test isolate and not in other samples sequenced on the same run. Together these indicated sensitive and specific detection of nematode DNA in our sample.

Phylogenetic analysis and global comparison

To contextualize the organism sequence obtained in this study, phylogenetic analyses were conducted by comparing representative reference sequences retrieved from GenBank.

These included *Dirofilaria* isolates originating from diverse geographic regions, including North America, Europe, and Asia (**Appendix Table 1**). Reference sequences were selected to capture both species-level diversity (e.g., *D. immitis, D. repens, D. tenuis, D. ursi, D. asiatica*) and geographic breadth based on available metadata. Multiple sequence alignments were performed to confirm speciation, and phylogenetic trees were inferred via neighborhood joining using Geneious.

Appendix Table 1. Summary of the GenBank accession numbers used in phylogenetic analyses, species, geolocation and source or host organism.

Gene	Accession	Species	Geolocation	Host
COX1	PV473791	*D. ursi*	Japan	Black bear
COX1	PX315770	*D. striata*	USA (Texas)	Dog
COX1	PQ219693	*D. tenuis*	Mexico	Raccoon
COX1	KT716014	*D. immitis*	Romania	Jackal
COX1	PV469768	*D. immitis*	Canary Islands	Dog
COX1	PX640822	*D. immitis*	Kosovo	Mosquito
COX1	PX946202	*D. repens*	Poland	Human
COX1	PV258722	*D. repens*	USA (Virginia) via Slovakia	Dog
COX1	PV400734	*D. repens*	Bosnia and Herzegovina	Mosquito
COX1	PV523836	*D. asiatica*	Sri Lanka	Dog
COX1	PP158772	*D. asiatica*	Bhutan	Dog
ITS2	PV382171	*D. ursi*	Japan	Bear
ITS2	EU182330	*D. immitis*	People’s Republic of China	Red panda
ITS2	EU182329	*D. immitis*	People’s Republic of China	Dog
ITS2	AF217800	*D. immitis*	Taiwan	Dog
ITS2	PX693401	*D. asiatica*	Sri Lanka	Dog
ITS2	JQ039743	*D. repens*	India	Dog
ITS2	JQ039744	*D. repens*	India	Dog
ITS2	PQ248143	*D. tenuis*	Mexico	Raccoon
28S	PV389592	*D. ursi*	Japan	Black bear
28S	KP760376	*D. repens*	Poland	Dog
28S	PX693401	*D. asiatica*	Sri Lanka	Dog
28S	KY990015	*D. immitis*	Mexico	Dog
28S	KP760375	*D. immitis*	Germany	Dog
28S	PQ248142	*D. tenuis*	Mexico	Raccoon
